# Incidentalomas during imaging for primary hyperparathyroidism—incidence and clinical outcomes

**DOI:** 10.1186/s12957-015-0687-2

**Published:** 2015-09-17

**Authors:** P. Prasad, C. Clout, E. Lorenz, B. J. Harrison, S. P. Balasubramanian

**Affiliations:** Endocrine Surgery Unit, Department of General Surgery, Sheffield Teaching Hospitals NHS Foundation Trust, Sheffield, UK; Department of Radiology, Sheffield Teaching Hospitals NHS Foundation Trust, Sheffield, UK; Academic Unit of Surgical Oncology, Department of Oncology, School of Medicine and Biomedical Sciences, The University of Sheffield, Sheffield, S10 2RX UK

**Keywords:** Parathyroid gland, Primary hyperparathyroidism, Imaging, Incidentalomas

## Abstract

**Background:**

Imaging for pre-operative localisation of parathyroid glands in primary hyperparathyroidism is now routine. This has led to the detection of incidental lesions (incidentalomas) in other organs, the nature of which is not well characterised.

The aim of this study was to determine the incidence, characteristics and outcomes in patients who had incidental findings on parathyroid imaging.

**Methods:**

Records of patients who underwent imaging for primary hyperparathyroidism over 2 years were reviewed to identify incidental lesions detected on parathyroid imaging. Patients with persistent or renal hyperparathyroidism were excluded. Details on the management of detected incidentalomas were obtained from patient records.

**Results:**

Incidentalomas were identified in 17 of 170 patients (10 %) undergoing parathyroid imaging. Incidentalomas included thyroid (*n* = 11), breast (*n* = 3), lateral compartment of the neck (*n* = 1), lung (*n* = 1) and clavicle (*n* = 1). However, no disease of clinical significance needing treatment was detected on further investigation.

**Conclusions:**

Although a significant proportion of patients undergoing parathyroid imaging had incidental lesions detected, these seem to be of little clinical significance. The morbidity and cost of further interventions on these incidentalomas need to be weighed against the benefits of routine imaging in improving outcomes of first-time surgery in patients with primary hyperparathyroidism.

## Background

Primary hyperparathyroidism (pHPT) is a common cause of hypercalcaemia and the third most frequently diagnosed endocrine disorder [1–3]. It occurs as a result of one or more overactive parathyroid glands; single-gland disease accounts for the majority of cases [2–5]. Whilst bilateral neck exploration used to be the mainstay of surgical treatment, minimally invasive surgical options are increasingly adopted [3–7]. Minimally invasive surgery relies on accurate pre-operative localisation of the abnormal gland(s). Technetium^99^ sestamibi (Tc99m MIBI) and cervical ultrasound (USS) are the most widely used investigations for pre-operative parathyroid localisation.

The use of pre-operative imaging in pHPT has led to the increased detection of incidentally detected lesions or ‘incidentalomas’ in other organs. For example, ultrasound can detect co-existing thyroid nodules as the thyroid gland is routinely evaluated in parathyroid imaging [5, 8]. Detection of these and other lesions elsewhere could lead to further investigations and surgery. The benefits of these interventions to the patient are debatable. Moreover, the results of imaging studies have the potential to delay parathyroid surgery and this may contribute to increased morbidity.

A series of such incidental lesions detected at this institution led to the initiation of this study. The aim of this study was to determine the incidence, management and clinical outcomes of incidentally detected lesions identified during pHPT imaging.

The objectives of the study were as follows:

1. Determine the incidence of incidentalomas during imaging for pHPT in a tertiary care institution over a 2-year period.
2. Identify the management strategies adopted and clinical outcomes following treatment of these incidentally detected lesions.
3. Evaluate any differences in length of waiting for parathyroidectomy amongst patients with and without incidentalomas.

## Methods

This was a retrospective cohort study. All patients who had a Tc99m MIBI and/or USS performed for parathyroid localisation prior to first-time surgery for pHPT between January 2010 and December 2011 at Sheffield Teaching Hospitals NHS Foundation Trust were included in this study.

The reports of Tc99m MIBI and USS scans in patients with pHPT were reviewed by one author (PP). Patients undergoing parathyroid imaging for persistent pHPT or renal hyperparathyroidism and patients who had undergone imaging in other hospitals were excluded. Any uncertainties regarding eligibility for inclusion into the study were discussed with the lead author (SPB). Data on the identification and lateralisation of enlarged glands and on the presence and nature of any incidental lesions identified was collected from radiology reports. A new finding unrelated to parathyroid pathology was deemed to be an ‘incidentaloma’ based on the reporting radiologist’s interpretation of the finding and on whether he/she had noted sufficient concern to warrant further investigation.

Further information on investigations performed for the incidentalomas, the results of these investigations, management strategies, relevant clinical outcomes and time taken from initial parathyroid imaging to surgery was retrieved from electronic and/or paper records. The Mann-Whitney *U* test was utilised to determine if there was a statistically significant difference in waiting times between patients with and without incidentalomas.

The proposal for the study was approved by the trust’s Clinical Effectiveness Unit.

## Results

**Fig. 1:**
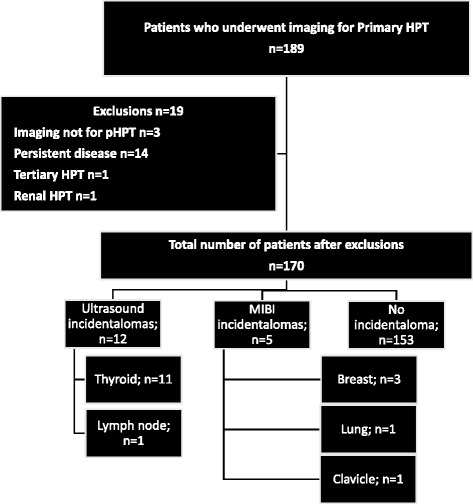
Flow chart depicting the flow of patients in this study. Out of 189 patients, 19 were excluded as they did not meet the inclusion criteria for the study. Out of the 170 patients included in the study, incidentalomas were detected on ultrasound (n = 12) or MIBI (n = 5) alone. Sites of the incidentalomas include the thyroid gland (n = 11), breast (n = 3), lymph node (n = 1), clavicle (n = 1) and lung (n = 1). There was no concordance between the imaging modalities for any incidental lesion identified

One hundred eighty-nine patients underwent parathyroid gland localisation scans between January 2010 and December 2011. Figure 1 demonstrates the process of inclusion of patients and the number and type of incidentalomas detected in this study.

The mean (standard deviation) age of the patients included in this study was 61(15.7) years. Most patients were females (114 or 67.1 % of the study population).

Seventeen of the 170 patients (10 %) undergoing imaging for pHPT had incidental lesions identified. Twelve of these were identified on ultrasound, and five were detected by a Tc99m MIBI scan. There was no concordance between the imaging modalities for any incidental lesion identified.

Eleven patients had thyroid nodules incidentally detected by ultrasound, that radiologists suggested required further investigation or monitoring. Six of these patients underwent an USS-guided fine-needle aspiration (FNA) of the thyroid nodule. Cytology from the FNA was benign in five patients and indeterminate in one patient. The patient with the indeterminate result had a lobectomy, the histology of which was benign. A total of four patients had the thyroid gland examined macroscopically at parathyroidectomy and had nodules excised or a lobectomy performed. Table 1 summarises the histological findings from the excised thyroid tissue in the five patients (macroscopic thyroid gland examination *n* = 4; lobectomy for indeterminate thyroid nodule *n* = 1) who underwent surgery. One patient had a 1.5-mm papillary microcancer detected histologically in a specimen that was predominantly a colloid goitre. One patient with a thyroid incidentaloma was managed conservatively; a follow-up ultrasound did not demonstrate any change to the incidental thyroid lesion.

**Table 1:** Histology results of surgically excised thyroid tissue

Histology	Number of patients
Florid Hashimoto’s thyroiditis	1
Moderate thyroiditis	1
Multinodular goitre	1
Colloid goitre with incidental 1.5-mm papillary microcancer	1
Benign appearance of thyroid tissue	1

Data illustrates the histology results of the thyroid tissue excised during surgery for primary hyperparathyroidism

Of the three patients with incidental breast lesions on MIBI scan, one patient had a breast ultrasound, one patient had a mammogram and one patient underwent both forms of imaging. No malignant features were identified in any patient, and none were subjected to biopsy.

Three patients had lesions detected elsewhere in the body. One patient had an enlarged cervical lymph node identified on ultrasound. Cytology from ultrasound-guided aspiration showed low-grade lymphoproliferative disease. As the patient was already under the care of haematologists for indolent non-Hodgkin’s lymphoma at the time of the scan, no further action was taken. The second patient had a lesion identified in the right lung on MIBI scan. CT thorax/abdomen and a PET scan confirmed an indeterminate lung nodule, thought to be a neuroendocrine tumour. The patient is being monitored by annual imaging. The third patient had increased uptake in the left clavicle on MIBI and underwent an X-ray. This demonstrated a normal bony appearance, and no further investigation or treatment was performed.

The median (interquartile range) waiting time for parathyroid surgery for patients with and without incidentalomas was 6.4 (3.5–7.0) and 5 (3.1–7.3) months, respectively; this was not statistically significant (*p* = 0.398).

## Discussion

Advancements in modern imaging and surgical techniques have enabled surgeons to adopt a minimally invasive approach in the treatment of many patients with pHPT [3, 9]. The success of such surgical approaches is largely dependent on accurate pre-operative localization of the overactive parathyroid gland(s) [7, 10]. One of the disadvantages of imaging is the detection of incidental lesions elsewhere in the body. Such lesions may require further investigation and treatment, potentially predisposing them to increased morbidity and delaying treatment of the primary condition for which imaging was requested. In the setting of parathyroid gland imaging for adenoma localisation, there is a gap identified in the literature on the incidence and risks of incidentalomas, nature of such lesions, their investigation and management.

The 10 % incidentaloma rate reported in our study is likely to be an underestimate. The vast majority of reporting was done by experienced consultant radiologists who have a special interest in parathyroid imaging. Thyroid nodules are commonly detected during ultrasound; however, only those that had equivocal or suspicious findings on imaging were highlighted by the radiologist and investigated further. Similarly, for MIBI reports, uptakes within physiological limits at other sites were not considered to be ‘incidentalomas’. Despite the careful selection of ‘incidentalomas’ in this study, the results have demonstrated that there was no pathology of major clinical significance identified on further assessment. Whilst the cohort size is small, the benign nature of the incidentalomas detected in this study may help reassure both patients and clinicians that most of these lesions do not tend to be of clinical concern.

Ultrasound for parathyroid localisation has led to the detection of concomitant thyroid pathology. The incidence, nature and suggestions for management of the incidentally detected thyroid nodule during parathyroid imaging have been well documented in the literature [5, 8, 11, 12]. However, there has been no study to our knowledge that examines the incidence, nature and management of extra-cervical incidentalomas identified during pHPT imaging.

### Thyroid incidentalomas

Ogawa et al. [11] have highlighted the high prevalence of thyroid disease in patients with hyperparathyroidism; 10.6 % of their patients had malignant thyroid tumours detected incidentally on ultrasound. A prospective cohort study by Adler et al. in 2010 [5] investigated the presence of thyroid pathology amongst 310 patients undergoing ultrasound for pHPT. Of the study population, 29 % had an incidental thyroid lesion identified at the time of ultrasound. The study demonstrated that approximately 2 % of all patients with pHPT undergoing an ultrasound had a thyroid malignancy.

In our study, only 6.5 % had a thyroid nodule that merited further imaging or intervention. Only one patient had an incidental papillary microcancer on histology within a colloid nodule detected on an ultrasound scan; this is considered insignificant.

### Breast incidentalomas on nuclear imaging

None of our three patients who had investigations for increased tracer activity in the breast had a clinically significant pathology identified. There have been reports of incidentally detected breast malignancies identified on imaging. For example, a study of suspicious breast findings in 902 women undergoing PET or CT scans identified 5 patients with a breast malignancy [13]. There are also reports that describe the detection of occult breast cancers in patients undergoing imaging with Tc99m MIBI [14–16].

### Other incidental lesions

Pulmonary nodules are incidental lesions that are frequently identified on imaging for other clinical indications. The subsequent management and follow-up of these lesions is variable and largely dependent on the nodule size, imaging modality and associated clinical features [17–19]. Wu et al. [20] suggest that small pulmonary nodules in patients less than 50 years old with no history of malignancy are unlikely to be of clinical significance. The literature describes the use and high specificity rates of nuclear imaging, particularly Tc99m MIBI scans, in the evaluation and management of solitary pulmonary nodules [21].

Our study identified the presence of a pulmonary nodule on the MIBI scan performed in one patient, necessitating further investigations.

Our case series identified one patient with increased bony uptake at the time of MIBI; this was localised to the clavicle. Qui et al. [22] have described the detection of vertebral metastases on a MIBI scan, in a patient with a rare parathyroid carcinoma. Zhao and Wang [23] have highlighted the relation between a high level of intact PTH and bone uptake of MIBI, concluding that this could reflect a stage of metabolic bone disease.

### Limitations

The results of this study are limited by the retrospective nature of the study design, the variability in the management of detected incidentalomas and the size of the cohort. A much larger sample size has the potential to detect clinically significant incidental lesions on parathyroid imaging. A multicentre study over a longer period of time could yield more precise estimates of the detection rate of lesions of clinical significance. This would allow a comprehensive study of the management practices adopted by different institutions, analyse the outcomes of the utilised management strategies and assist in the development of an appropriate management algorithm for such incidentalomas.

## Conclusions

The incidence of incidental lesions amongst patients undergoing localisation studies for pHPT was 10 %. The clinical significance of these lesions is low. Although larger studies are required to assess the true clinical impact of detection of these incidentalomas, the results of this study serve to provide initial data that addresses the knowledge gap regarding the incidence and nature of these incidental lesions.
